# Shared and unique patterns of autonomous human endogenous retrovirus loci transcriptomes in CD14 + monocytes from individuals with physical trauma or infection with COVID-19

**DOI:** 10.1186/s12977-024-00652-z

**Published:** 2024-11-04

**Authors:** Hyunmin Koo, Casey D. Morrow

**Affiliations:** 1 Department of Genetics Hugh Kaul Precision Medicine Institute, University of Alabama at Birmingham, Birmingham, Alabama, United States of America; 2 Department of Cell, Developmental and Integrative Biology Hugh Kaul Precision Medicine Institute, University of Alabama at Birmingham, Birmingham, Alabama, United States of America

**Keywords:** Autonomous HERV transcriptome, CD14+monocytes, ScRNA-seq, Pangenome, Innate immunity, COVID-19

## Abstract

Since previous studies have suggested that the RNAs of human endogenous retrovirus (HERV) might be involved in regulating innate immunity, it is important to investigate the HERV transcriptome patterns in innate immune cell types such as CD14 + monocytes. Using single cell RNA-seq datasets from resting or stimulated PBMCs mapped to 3,220 known discrete autonomous proviral HERV loci, we found individual-specific variation in HERV transcriptomes between HERV loci in CD14 + monocytes. Analysis of paired datasets from the same individual that were cultured in vitro with LPS or without (i.e. control) revealed 36 HERV loci in CD14 + monocytes that were detected only after activation. To extend our analysis to in vivo activated CD14 + monocytes, we used two scRNA-seq datasets from studies that had demonstrated activation of circulating CD14 + monocytes in patients with physical trauma or patients hospitalized with COVID-19 infections. For direct comparison between the trauma and COVID-19 datasets, we first analyzed 1.625 billion sequence reads from a composite pangenome control of 21 normal individuals. Comparison of the sequence read depth of HERV loci in the trauma or COVID-19 samples to the pangenome control revealed that 39 loci in the COVID-19 and 11 HERV loci in the trauma samples were significantly different (Mann-Whitney U test), with 9 HERV loci shared between the COVID-19 and trauma datasets. The capacity to compare HERV loci transcriptome patterns in innate immune cells, like CD14 + monocytes, across different pathological conditions will lead to greater understanding of the physiological role of HERV expression in health and disease.

**Supplementary Information:**

The online version contains supplementary material available at 10.1186/s12977-024-00652-z.

## Introduction

Approximately 8% of the human genome consists of human endogenous retroviruses (HERVs) [1]. Many HERVs contain both 5’ and 3 long terminal repeats (LTRs) that can promote transcription [2, 3]. Some of these LTRs are solo LTR sequences that are shorter in length and have the potential to promote the transcription of neighboring host genes [4–8]. Jacques et al. found that a disproportionate number of LTRs are present in cell type-specific DNase I hypersensitive sites, indicating accessible chromatin regions for transcription [9]. Autonomous LTRs contain nearly full-length proviral transcripts that encode *gag*,* pol*,* en*v, and, sometimes, additional accessory genes [3, 10, 11]. Even though most do not produce complete proteins due to the accumulation of mutations over the course of evolution, there is a growing recognition that these HERV RNA transcripts may play a role in the innate host response [12, 13].

Further understanding of the expression of the autonomous proviral HERV transcriptome in both normal and disease states is needed to evaluate their contribution to the networks of gene expression in the host [14]. One of the challenges in characterizing the HERV transcriptome is the number and lack of heterogeneity of HERV genes, which makes it difficult to distinguish changes in transcription [10]. To circumvent this issue, previous studies have identified a dataset characterizing the position and gene content of approximately 3220 individual proviral HERV loci [3, 10]. Several studies have used this dataset to map HERV loci in cell lines and primary cells, showing cell type specific HERV loci transcriptomes [3, 10, 15–17]. Additional analysis using RNA-seq databases reported that the HERV loci transcriptome patterns in peripheral blood mononuclear cells (PBMCs) from patients with COVID-19 differed from those of uninfected patients, leading to the suggestion that the expressed HERV loci could act as sentinels for the innate host response to viral infections [12].

Human PBMC consists of a consortium of immune system cells that include, at a minimum, T cells, B cells, monocytes (CD14+), dendritic cells and natural killer cells [18, 19]. Given that the analysis of active HERV loci transcription is dependent on cell type, using specific subsets of PBMCs could provide new insights into HERV expression in these immune cells. Since CD14 + monocytes are a major cell type involved in innate immunity, it is important then to compare the expression patterns of HERV transcription in these cells between normal individuals and patients with perturbations known to stimulate CD14 + monocytes [20–22]. In the current study, we downloaded publicly available PBMC-related single-cell RNA sequencing (scRNA-seq) datasets and analyzed them to identify HERV loci and compare HERV profiles among samples. For the analysis, we utilized existing informatics tools such as Seurat [23] and 10X Cell Ranger [24] and developed our informatics tool to cluster DNA sequences at 99% identity and map them onto 3,220 specific HERV loci in CD14 + monocytes. Using this pipeline, we characterized the impact of PBMC isolation methodology on the detection and reproducibility of identified HERV loci transcriptomes. We then analyzed the HERV loci transcriptomes from studies that have shown activated CD14 + monocytes: PBMC activated in vitro by LPS and PBMC from separate datasets of patients with physical trauma or from patients hospitalized with COVID-19 [25, 26]. We also developed a pangenome control RNA-seq dataset consisting of a composite of 21 normal individuals to identify HERV loci transcriptomes that were shared or specific in the host following trauma or COVID-19 infection. The identification of shared and unique HERV loci transcriptome patterns in CD14 + monocytes between individuals with different pathological conditions will provide new perspectives on the role of CD14 + monocyte HERV transcriptome expression in the innate immune response.

## Results

**Fig. 1:**
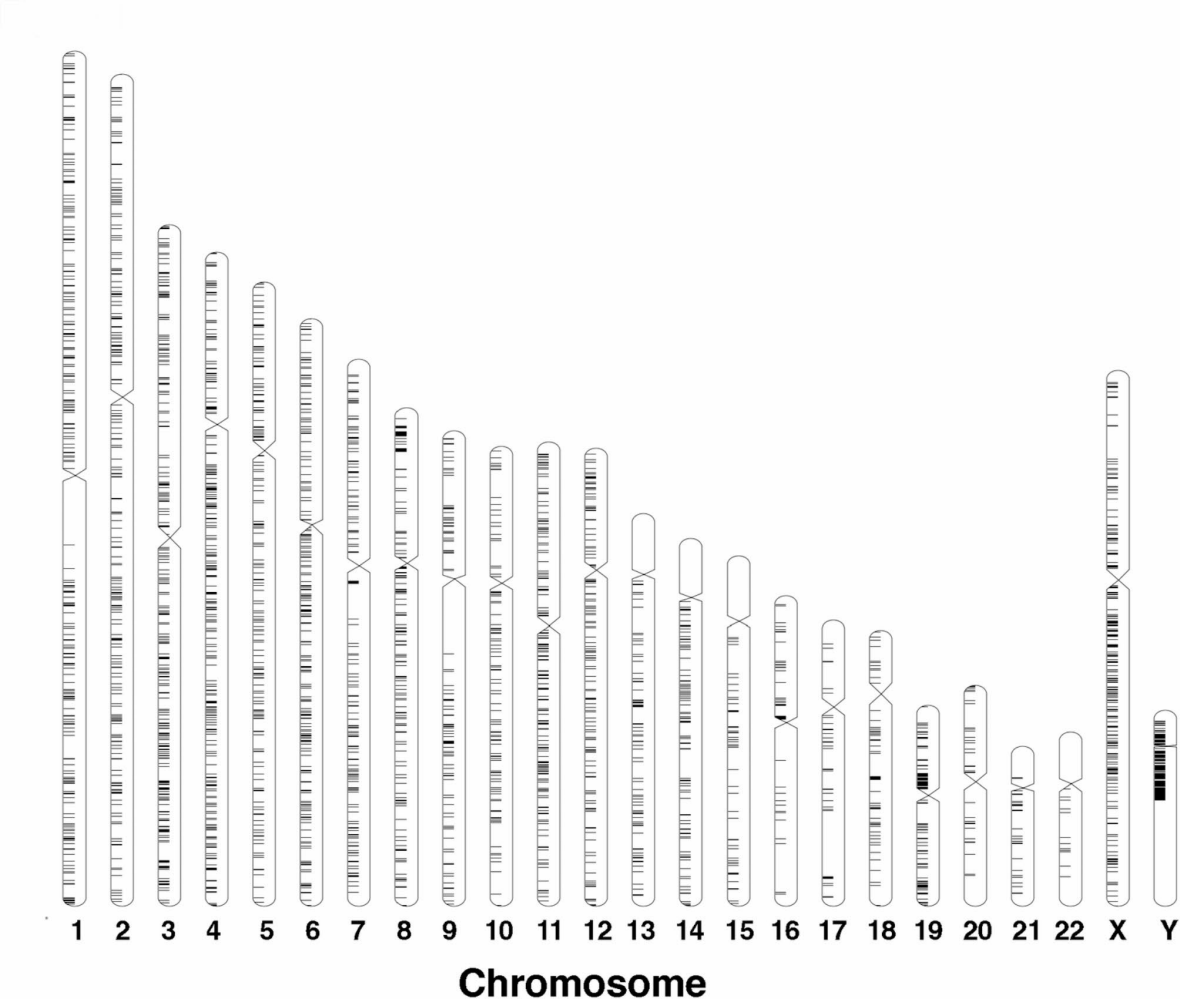
Distribution of HERV proviral loci in human chromosomes

Previous studies have identified 3,220 autonomous proviral HERV loci throughout the human genome that are distributed throughout the 23 human chromosomes [3, 10] (Fig. 1, Supplementary Table 1). Most of these proviral HERV genomes contain intact 5’ and 3’ long terminal repeats that would promote transcription with a disproportionate number of LTR‘s located in accessible chromatin regions for transcription [3, 9]. However, previous studies examining the HERV transcriptome have demonstrated that only a fraction of the HERV loci are expressed under various physiological conditions and diseases [10–12, 15, 27–29]. Therefore, to understand the role of HERV transcription in normal and pathological conditions, it is important to compare the modulation of individual HERV loci transcriptomes across different pathological conditions.

In the current study, we modified our previous informatic method to align 3,220 HERV loci for each scRNA-seq dataset to determine the common or unique transcription patterns in HERV loci (Supplemental Methods [30]), . The new method, called Window-based HERV Alignment (WHA), compares aligned DNA sequences in pairs using sequential, non-overlapping windows of defined nucleotide lengths 99% alignment requirement. Thus, our positive designation of a HERV loci transcriptome reflects increased transcription, analogous to a positive differential gene expression, as described for the 3,220 HERV loci [10, 12, 15]. HERV loci designated as negative do not meet the required read depth or number of usable (good) windows as determined by WHA.

**Fig. 2:**
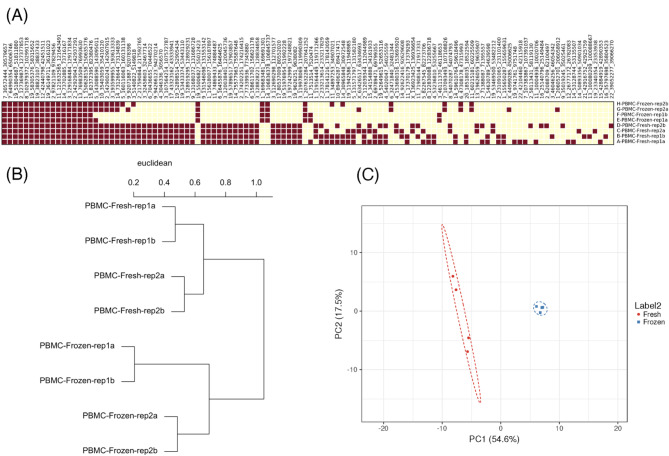
Impact of PBMC preparation on the detection of positive HERV loci. A scRNA-seq dataset of paired PBMC samples from the same donor that were prepared from fresh (not frozen) and those samples from the same donor that were frozen prior to use [26]. We sorted sequences for CD14 + monocytes and analyzed output file to conduct the WHA. **A** Heatmap of positive HERV loci detected from PBMC. Individual specific transcribed HERV loci the repeat samples (rep1A, rep1B, and rep2A rep 2B) are presented. The percent similarity for the paired fresh samples: 82.5–84.2% and frozen samples: 92.9–100%. **B** Phylogenetic tree of the PBMC prepared fresh after isolation versus frozen. phylogenetic tree shows the individual similarity of the repeat samples and differences between fresh and frozen from the same individual. **C** Clustering of positive HERV loci from fresh or frozen sample sets. A PCA of illustrates the clustering between the fresh and frozen samples and highlighting the individual specificity of the HERV loci identification

A unique feature of our study is the use of WHA on scRNA-seq datasets combined with informatic tools to identify the transcriptomics of unique HERV loci in CD14 + monocytes (Supplementary Fig. 1) [23]. To characterize this analysis, we selected a unique scRNA-seq dataset of CD14 + monocytes from paired PBMC samples from the same donor that were prepared from fresh (i.e. not stored) and those samples from the same donor that were frozen before use [26] (Fig. 2). We identified individual specific transcribed HERV loci from the repeat samples from the same individual (rep1A, rep1B, or rep2A rep 2B). A heatmap was used to depict the HERV abundances that were positive. We found that individual samples prepared in the same way (either fresh or frozen) had a similar distribution of HERV loci (Fresh samples: 82.5–84.2% and Frozen samples: 92.9–100%) (Fig. 2A). Using a phylogenic tree analysis, we found that the individual samples (1 and 2) prepared the same way (fresh or frozen) were on separate branches of the phylogenetic tree within the same branch, and the individual samples were located on different branches of the phylogenetic tree (Fig. 2B). Finally, the PCA analysis revealed a difference in the clustering between the fresh and frozen samples. Interestingly, we found that the individual specificity of the HERV loci for 1 and 2 was more apparent in the fresh samples, while both individuals in which samples were prepared frozen samples clustered together (Fig. 2C). To ensure that this clustering was not due to differences in sample read numbers, we repeated the analysis with equal read numbers per sample (randomly subsampled) and confirmed that there were no changes in the phylogenetic tree or PCA plot following the random subsampling process (Supplementary Fig. 2). The results show that the reproducibility of the analysis and that the HERV loci transcriptome is individual specific and sensitive to the method of PBMC sample preparation.

To determine if our analysis protocol could be used to detect HERV loci from activated CD14 + monocytes, we next used a second dataset in which a single individual PBMC was divided into 4 sample sets in which one pair served as a control and a second pair was stimulated with LPS, a known stimulant for CD14 + monocytes [20, 26]. All 4 PBMC datasets of individual scRNA-seq were analyzed and the sequence reads for CD14 + monocyte reads were subjected to WHA analysis. We filtered the WHA results to identify HERV loci that were expressed in activated but not resting cells. For this result, we presented only those loci that were the pair from LPS stimulated samples that were both positive for transcription. We identified 36 HERV loci in CD14 + monocytes that were detected only after stimulation with LPS (Supplementary Table 2). Six chromosomes (2, 3, 4, 10, 12 and 17) were identified with the greatest numbers of HERV loci that were differentially expressed in activated cells (Supplementary Table 3). Chromosome 2 had the most identified HERV loci that were expressed in the activated but not resting CD14 + monocytes. We also found that these sites were surrounded by HERV loci that were not expressed following LPS stimulation. Overall, the results from this paired sample dataset establish the utility of our analysis to detect HERV loci transcription from resting and in vitro (LPS) activated CD14 + monocytes.

**Fig. 3:**
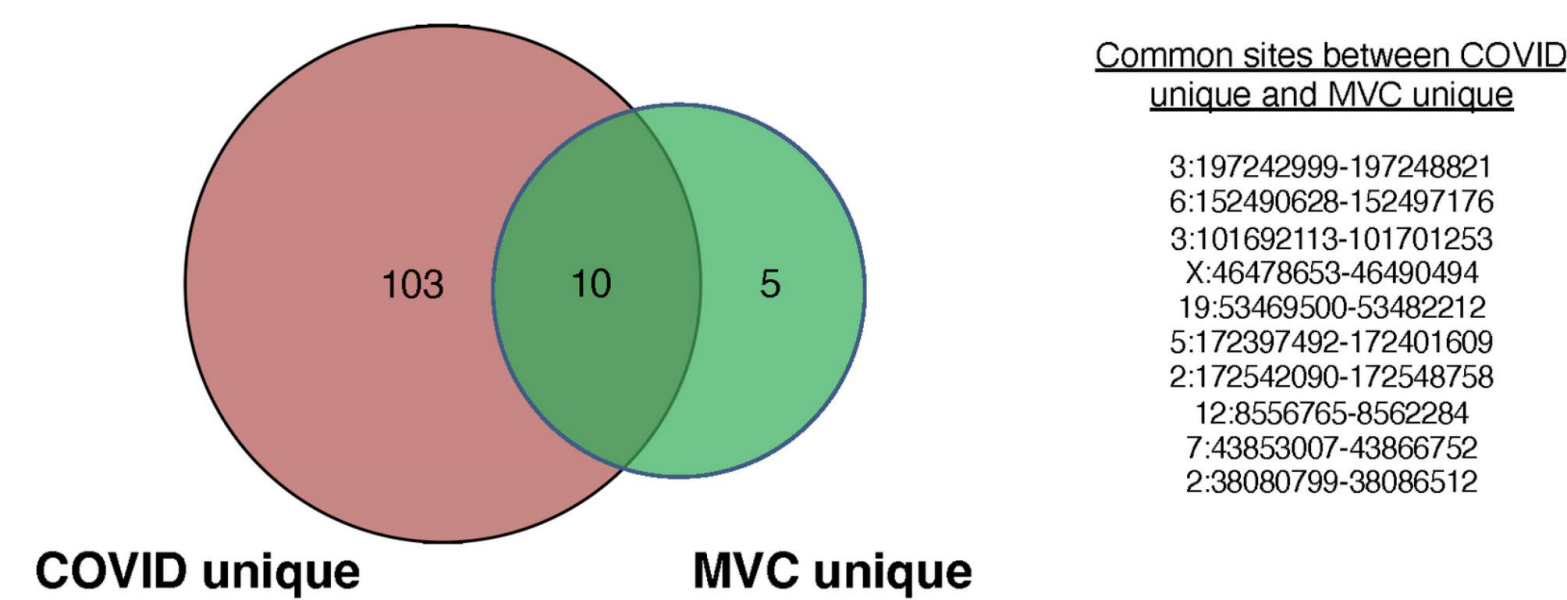
Venn diagram of positive HERV loci in CD14 + monocytes from samples obtained from patients after physical trauma or hospitalized from COVID-19. Two scRNA-seq datasets from studies where in vivo activation of CD14 + cells were used: one study that examined the impact of physical trauma while a second study examined patients hospitalized with COVID-19 infections [25, 32] (Supplemental Table 4). A Venn diagram was generated to depict the similarities and differences between the HERV loci detected from each study. From the trauma dataset, 15 discreate HERV loci in patients with physical trauma that were not found in the 10 samples from control individuals. From the COVID patients, 113 discreate loci that were not found in the 6 control subjects (Supplemental Table 5). 10 of the transcribed HERV loci were shared between the trauma and COVID-19 sample sets. The identification of the sites is presented in Supplemental Table 5. The 10 HERV loci that were shared between the two studies are also presented

A recent study described an extensive database called DISCO (Deeply Integrated Single-Cell Omics data) that allows large-scale integration of scRNA-seq data [31]. Many of the studies in DISCO are in PBMC that analyzed frozen PBMC that did not undergo in vitro stimulation. To extend our analysis of the HERV transcriptome, we used two extensive scRNA-seq datasets from studies where in vivo activation of CD14 + cells was found; one study examined the impact of physical trauma while a second study examined patients hospitalized with COVID-19 infections [25, 32] (Supplementary Table 4). Both studies included PBMC isolated from normal individuals that served as controls. We first filtered the CD14 + monocyte sequences from the patients and controls associated with each study. We identified HERV loci that were not transcribed in the normal individual controls for each study but were transcribed in at least 1 of the patients (either physical trauma or COVID-19). We identified 15 discrete HERV loci in patients with physical trauma that were not found in the 10 samples from control individuals. From the COVID-19 patients, we identified 113 discrete loci that were not found in the 6 control subjects (Fig. 3). 10 of the transcribed HERV loci were shared between the trauma and COVID-19 sample sets (Fig. 3, Supplemental Table 5).

**Fig. 4:**
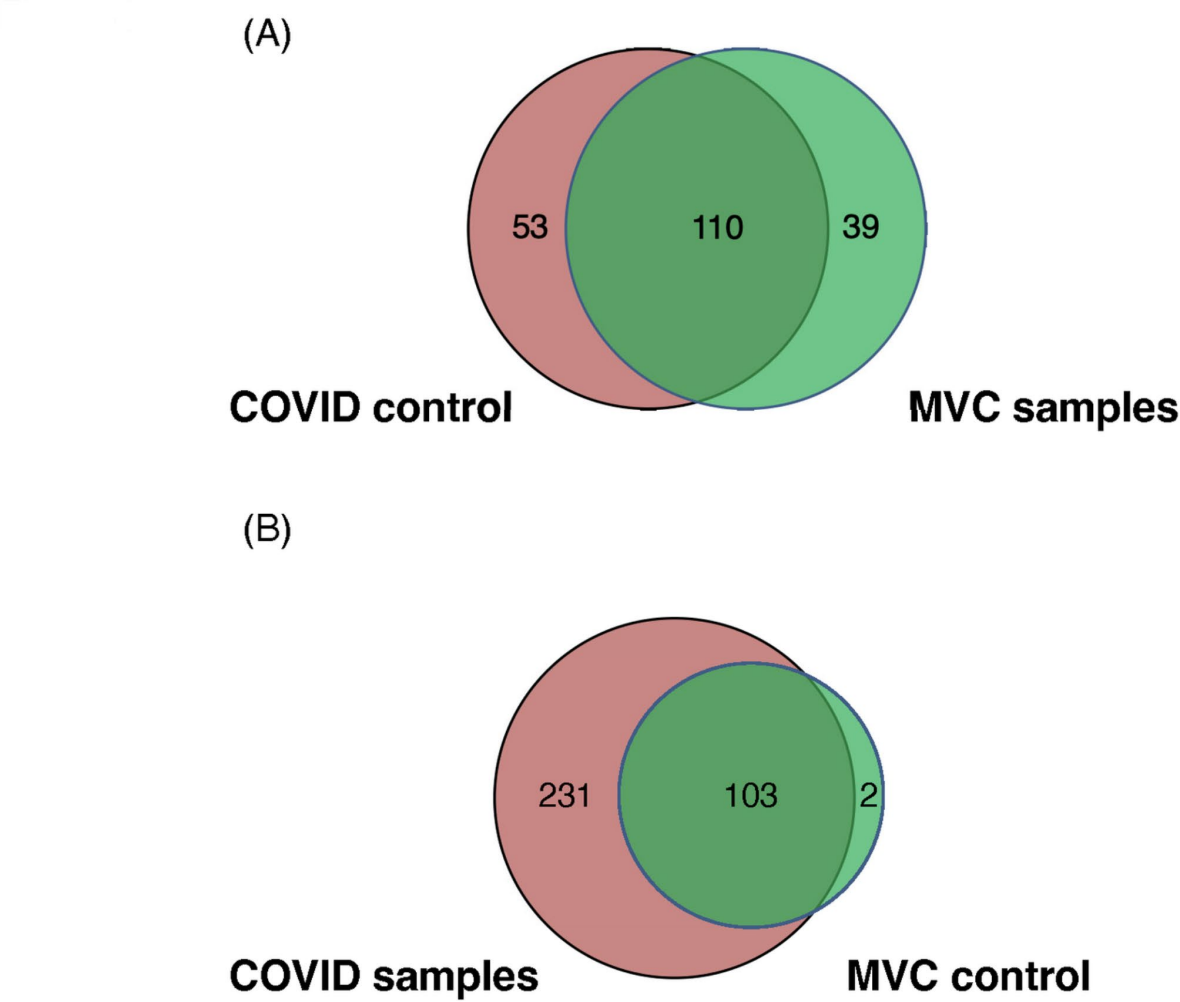
Venn diagrams of positive HERV loci identified from patients with trauma or hospitalized with COVID-19 using different control samples. The analysis of the transcriptome of HERV loci for the datasets presented in Fig. 4 used the specific control samples for each study. The trauma samples were filtered with the COVID-19 controls while the COVID-19 samples were filtered with the trauma control sample set. **A** The trauma samples were filtered against the COVID-19 controls and the numbers of unique and common HERV loci were identified. **B** The COVID-19 samples were filtered using the trauma control samples and the number of unique and common HERV loci sties were identified

A concern for using only the study specific normal individuals for controls is the variability between the numbers of individuals, and hence the total number of DNA sequence reads, for CD14 + monocytes between different studies. To illustrate this point, we compared the transcribed HERV loci of the COVID-19 and trauma samples with either the control samples from the trauma or COVID-19 study (Fig. 4, Supplementary Table 6). We found that the number of unique and shared HERV loci between the COVID-19 and trauma samples varied depending on the control dataset that was used. For example, the number of discrete HERV loci in the COVID-19 samples compared to the trauma samples was 53 if we only used the control samples from the COVID-19 dataset but 231 if we used the control dataset from the trauma dataset (Fig. 4). Similar differences in the pattern of unique HERV loci were identified in the trauma datasets, as well as in the shared HERV loci between the COVID-19 and the trauma datasets. The results highlight the dependency of HERV loci identification on the number of control samples used in the analysis.

**Fig. 5:**
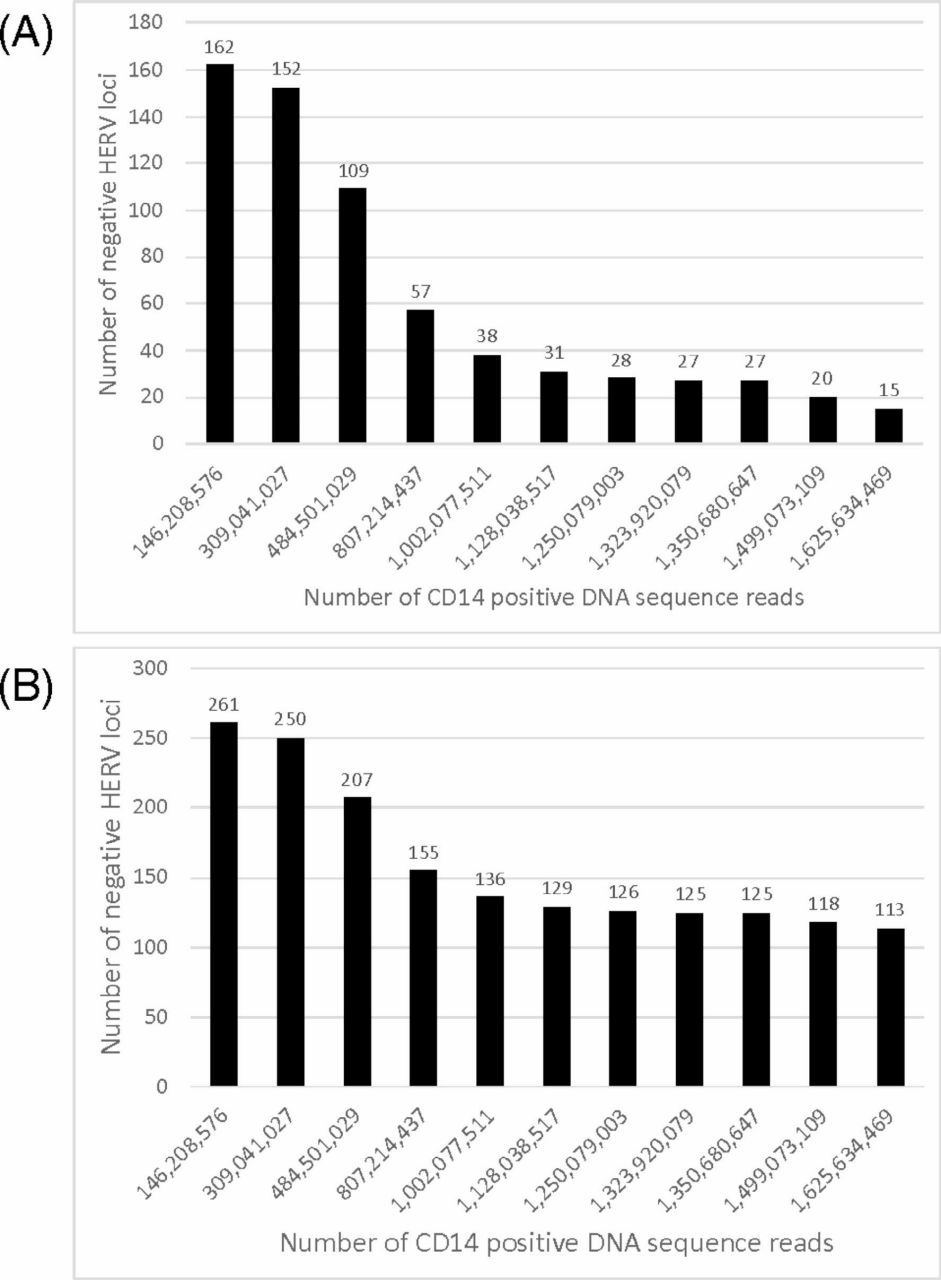
Pangenome control for identification of positive HERV loci. **A** For the composite 21 normal pangenome control versus the trauma dataset, we determined that 162 out of a possible 3,220 HERV loci were positive. From a plot of the negative HERV loci versus the number of DNA sequence reads, we found that the sequential addition of control samples up to 1.625 billion reduced the number of negative loci that leveled off at 15 HERV loci. **B** A similar strategy was employed for the composite 21 pangenome control panel versus the combined (live and dead) COVID-10 dataset. In this case, we determined out of a total of 261 loci with at least 1 positive comparing the pangenome with the COVID-19 dataset. After filtering the sequential addition of sequence reads up to 1.625 billion reads with the COVID-19 dataset we found the negative HERV loci leveled off at 113

To circumvent this issue, we established a composite pangenome of control samples consisting of the study controls plus an additional 5 normal PBMC samples giving a total of 21 normal human controls [33, 34]. Following WHA, we identified the negative and positive HERV loci of the 21 composite samples from normal individuals and the trauma or COVID-19 datasets (Supplementary Table 7). To further characterize this result, we found that the sequential addition of control samples up to 1.625 billion reduced the number of negative loci that eventually leveled off 15 HERV loci even with the addition of new reads from other control samples (Fig. 5A). We next used manual filtering of the Excel file in Supplementary Tables 7 to identify HERV loci transcriptomes only expressed in the trauma datasets (Supplementary Table 8). Interestingly, the 15 loci were found in 5 individuals who had a previous motor vehicle collision (MVC) while no positive HERV loci were found in the remaining 5 that did not have an identified MVC. A similar strategy was employed for the 21 pangenome of the control samples compared with the combined (live and dead) COVID-19 dataset. In this case, we determined the HERV loci detected leveled off at 125 unique HERV loci at 1.32 billion reads with the sequential addition of 300 million sequence reads up to 1.625 billion reads giving a final HERV loci of 113 (Fig. 5B). From the analysis of both the COVID-19 patient datasets, we found that 113 HERV loci were identified that were absent from the control (Supplementary Table 8).

For both the trauma and COVID-19 analyses we found individual specific patterns of the HERV loci transcriptomes expression. For the trauma patients, we noted individual specific patterns of the positive HERV loci with 1 individual with 8 loci and the rest of the individuals with 1–4 loci (Supplementary Table 8). We found the datasets from the living COVID-19 patients had 2 individuals with over 20 loci, 2 individuals with 12 loci, 1 individual with 9 loci, and the remaining 2 individuals with ranges from 4 to 8 loci. We found from analysis of the patients that died after 7 days had 1 individual with 46 loci, 1 individual with 18 and the rest of the individuals with 1–13 loci. The differences between the HERV loci transcriptome expression patterns in the trauma or COVID-19 samples versus the controls did not correlate with the DNA sequence read count (Supplemental Fig. 3).

**Fig. 6:**
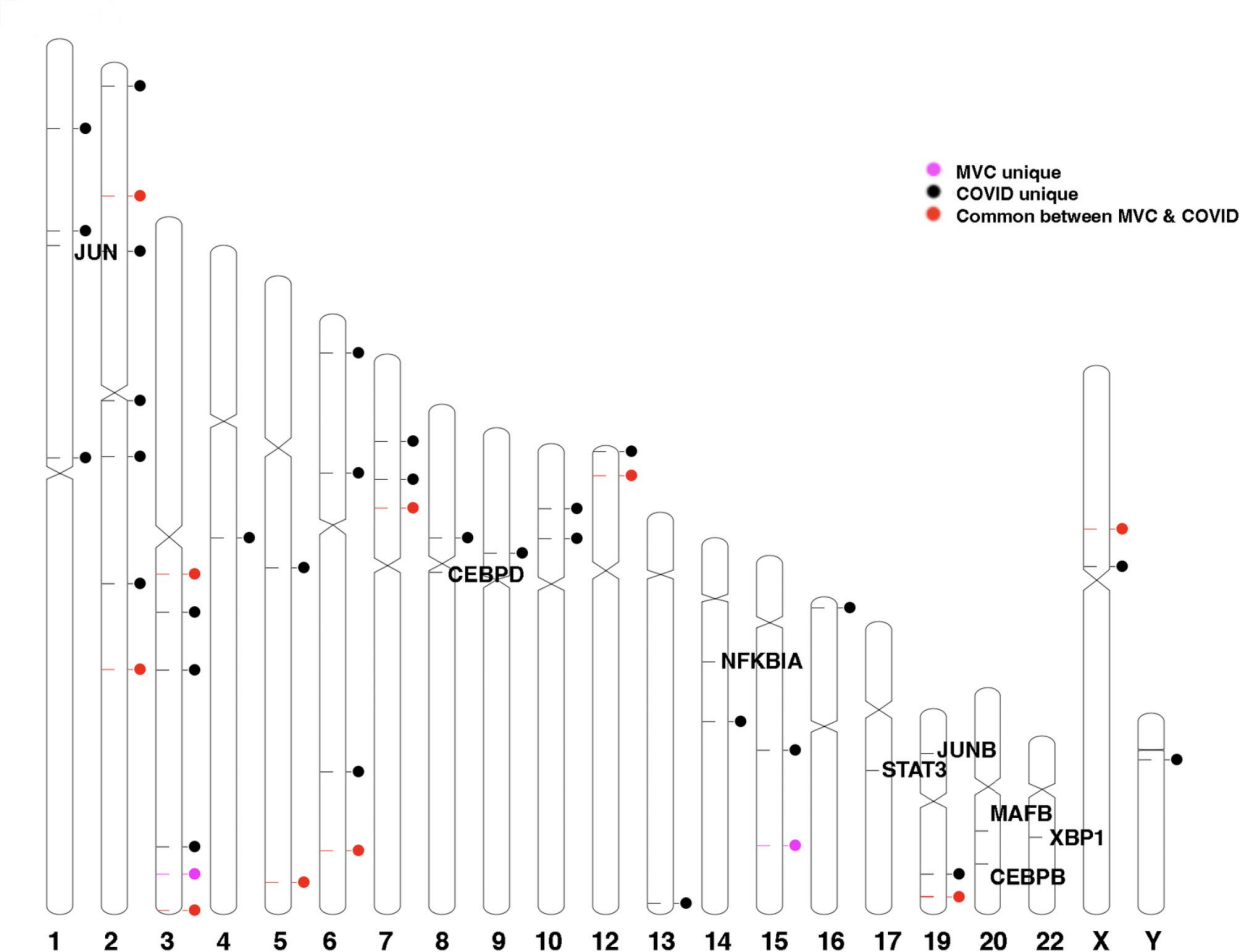
Chromosome distribution of positive HERV loci identified from the trauma or COVID-19 datasets. The distribution of the HERV loci in the 23 human chromosomes and expressed host cell genes in CD14 + monocytes were depicted using *Idiographica* [52]. We did not find a transcribed HERV loci in chromosomes 11, 18, and 21. Chromosomes 2 and 3 had the most HERV loci (range 4–7 loci/chromosome) while the remaining chromosomes had a range of 1–4 HERV loci per chromosome

Those HERV loci identified with 2 more plus were subjected to a statistical analysis comparing the sequence read depth with the pangenome control read depth at the same loci (Supplemental Tables 9 and 10). Using a Mann-Whitney U test, we found that all HERV with 2 or more positive identified in the 11 MVC and 39 COVID-19 HERV loci were different from the controls (*p* < 0.05). We identified 9 HERV loci that were shared between the trauma and COVID-19 patients. No shared pattern of predicted canonical HERVs between these shared 9 HERV loci was evident [3] (Supplemental Table 11). Since we used the same control dataset for the trauma and COVID-19 datasets, we mapped the distribution of the HERV loci over the 23 human chromosomes (Fig. 6). Chromosomes 2 and 3 had the most HERV loci (range 4–7 loci/chromosome) while the remaining chromosomes had a range of 1–4 loci (Fig. 6). The distribution of the HERV loci in the 23 chromosomes did not correlate with host genes identified in which transcription was significantly increased in activated CD14 + monocytes [25, 32].

## Discussion

In this study, we have examined the patterns of autonomous HERV loci transcriptomes in normal and activated cells. Using scRNA-seq datasets from PBMC, we have used informatic tools, Seurat and 10X Cell ranger to identify clusters of CD14 + monocytes. The DNA sequence reads from CD14 + monocytes were aligned to 3220 autonomous HERV loci to identify HERV loci transcriptomes. We demonstrated that the transcriptome pattern of HERV loci in CD14 + monocytes was specific for the individual and reproducible between paired samples prepared by the same method for scRNA-seq. We developed a composite 21 normal individual pangenome control panel to identify HERV loci that were preferentially expressed in activated, but not quiescent, CD14 + monocytes from patients that had undergone physical trauma and patients infected with COVID-19.

Previous studies have identified approximately 3,220 autonomous proviral HERV loci that are distributed throughout the 23 human chromosomes [3, 10]. Most of these autonomous proviral genomes contain intact 5’ and 3’ LTR that would promote transcription to express RNA [3]. The challenge for mapping of HERV loci transcription is that many of the HERV loci contain the same consortium of genes resulting in repetitive DNA from scRNA-seq [35]. Other studies have used different informatics techniques and methods to map transcribed RNA to the 3220 ERV loci in PBMC [3, 10, 15]. However, PBMCs are composed of several different immune cell types [18]. The results of our study are unique because we analyzed the transcriptome of HERV loci from CD14 + monocytes. To do this, we developed informatics tools used to identify specific cell clusters (Seurat and 10X Ranger) and adapted our previous informatics technique (WSS) to evaluate HERV loci transcriptomes from CD14 + monocytes [30, 36]. We then applied several filtering steps to identify those HERV loci that had increased transcription compared to the same HERV loci from the control sample. It is important to note that because of our sequence alignment constraints (i.e. 99% identity) and our filtering steps, our data reflects the patterns of the highest expressed HERV transcriptomes from the individual 3,220 proviral HERV loci compared to the same HERV loci of control samples from normal individuals.

To characterize our analysis pipeline, we analyzed the HERV loci transcriptomes from a unique scRNA-seq dataset that contained paired samples [26]. We found that the analysis was reproducible, as evident from the similarity of the patterns of the HERV loci transcriptomes between the paired samples of the same individual. The patterns of HERV loci transcriptomes were individual specific as evident from the comparison of the freshly prepared samples from two different individuals, a result that is consistent with a recent study that used a comprehensive single cell analysis to show immune cell diversity between individuals [19]. The processing of the PBMC samples can influence the number of transcribed HERV loci as evident from the different patterns of HERV loci transcriptomes from matched frozen and fresh, paired samples. Interestingly, the sample prepared from the paired frozen PBMC had fewer positives than the fresh samples. Previous studies have observed that freeze/thawing is known to damage cells, possibly leading to RNA instability [37–39]. Since the majority of the transcribed HERV loci do not encode RNA’s that are translated they might also be more susceptible to degradation [3]. The results of our studies then highlight the reproducibility of our analysis pipeline and the importance of using sample sets that have undergone the same method of sample preparation when comparing the patterns of the HERV loci transcriptomes.

The goal of our study was to identify HERV loci that could be used to discriminate resting from activated CD14 + monocytes. In the first step, we used a paired dataset using similarly processed control and LPS stimulated PBMC from the same individual [26]. LPS is known to be a potent stimulator of CD14 + monocytes, probably due to the cells having a receptor for LPS [21]. We identified 36 HERV loci in CD14 + monocytes that were detected only after stimulation with LPS. Six chromosomes (2, 3, 4, 10, 12 and 17) were identified with the greatest numbers of HERV loci that were differentially expressed in LPS activated cells.

We next extended our approach to analyze in vivo CD14 + monocytes from PBMC that were prepared by the same method (i.e. from frozen samples). Previous studies have found that trauma causes an abrupt transition from a healthy state to a system-wide physiological crisis characterized as a genomic storm in PBMC [32, 40, 41]. The dataset selected for our study was used by Chen et al. that found after systemic injury the gene expression pattern of CD14 + monocytes was different from control samples [32]. We identified 15 discrete HERV loci in CD14 + monocytes from patients with physical trauma that were not found in the 10 samples from control individuals. In a second study, Amrute et al. found that CD14 + monocyte transcription patterns were associated with and predicted survival from COVID-19 infection [25]. Using the scRNA-seq dataset from these COVID-19 patients, we identified 113 discrete loci in CD14 + monocytes that were not found in the 10 control subjects [25]. For both studies, we found that not all samples from all of the individuals in either study were HERV loci positive. Since a previous study has used single cell analysis to show that substantial diversity exists between the immune cell profiles of individuals, the distribution of positive HERV transcriptomes in the scRNA-seq datasets could reflect the differences in the physiologic status of the individual’s immune system [19].

We found that 10 of the transcribed HERV loci were shared between the trauma and COVID-19 sample sets using the normal control samples provided for each study. However, substituting the normal dataset from one study with another resulted in an altered pattern of the HERV loci transcriptomes indicating the HERV loci specific for the trauma or COVID-19 was dependent on the control dataset used for comparison. To circumvent this issue, we developed a composite pangenome control panel consisting of 21 normal individual pangenome control consisting of over 1.625 billion sequence reads that could be used to more directly compare the HERV transcription loci from a dataset of patients post physical trauma and the patients with COVID-19 [33]. Using this composite pangenome control, we found for the trauma patients 15 discrete ERV loci that were negative in the controls. Interestingly, the 15 loci were only found in 5 individuals who had a previous motor vehicle collision (MVC) while the remaining 5 with no positive HERV loci were found in those individuals with other trauma. The expression of HERV loci in CD14 + monocytes from patients who had MVC is consistent with previous studies that have found that physical trauma can increase gene expression in CD14 + monocytes, supporting future studies to extend this result using datasets from individuals following other traumas [41].

From the analysis of the COVID-19 patients, we found that 113 HERV loci were identified that were absent from the pangenome control. The fact that the COVID-19 samples had greater numbers of the HERV loci is consistent with the samples from patients who were hospitalized with COVID-19 infection, which is known to result in a strong immune response [32, 40–43]. Furthermore, we observed a trend that the hospitalized patients who subsequently died had greater numbers of the HERV loci with positive transcriptomes than those patients who survived, a result that is consistent with Brauns et al.. who found that severe COVID-19 infection has an impact on the differentiation status and function of circulating monocytes [44]. Grandi et al. also found differences with the HERV loci with respect to the status of the patients after COVID-19 infection [12]. A comparison of the HERV loci identified from our study and the Grandi et al. study revealed differences that could be accounted for by the source of the PBMC dataset since the Amrute et al. dataset used hospitalized patients while *Grandi et al.* study used convalescent patients. In addition, the Grandi et al. studies use datasets from PBMC consisting of T cells, B cells and monocytes and would be expected to be different from the CD14 + monocytes used for our analysis.

Finally, we identified 10 HERV loci that were common between the MVC trauma and the COVID-19 dataset when compared to the pangenome control. It is possible that the HERV transcriptome expression from these 10 loci identifies a core group of activated CD14 + monocytes that could be part of the trained immunity programs that have been identified in CD14 + monocytes [45]. In future studies to further explore this possibility, we could extend our approach to compare other immune-related diseases such as systemic lupus erythematosus or even cancer [10, 27–29]. To do this we use the 21-composite individual pangenome and even add to this panel with more control samples from the new studies. Comparison of specific patterns of HERV loci transcriptomes in CD14 + monocytes from patients with different immune related diseases then could provide insights that could lead to a greater definition of the role of HERV transcriptomes in the innate immune response [2, 13].

## Materials and methods

### Dataset used in this study

In this study, we used 4 publicly available scRNA-seq PBMC datasets from (1) 3 healthy individuals from Thompson, E.A., et al. [34], (2) 2 healthy individuals from Derbois et al. [26], (3) 6 healthy individuals and 12 critical COVID-19 patients who survived (*n* = 6) or died (*n* = 6) on both days 0 and 7 of study enrollment from Amrute et al. [25]. and (4) 10 healthy individuals and 10 trauma patients from Chen et al. [32]. Accession numbers for samples used in this study are below the Data availability section.

### scRNA-seq analysis

Publicly available scRNA-seq PBMC data were downloaded in either fastq or bam format, whichever is available from NCBI GEO or SRA. Each bam file was converted into fastq format using 10x Genomics Cell Ranger software (v 7.1.0) bamtofastq (v 1.4.1). All fastq files were then aligned to the human reference genome (GRCh38) using Cell Ranger count with default parameter settings. For each sample, CD14 + monocytes were selected based on matrixes files provided by primary publication, or matrixes (.rds format) from the Deeply Integrated human Single-Cell Omics data (DISCO) [31], or matrixes generated by the SingleR package using the Database of Immune Cell Expression (DICE) reference dataset [46]. Barcodes associated with CD14 + monocytes were listed in .txt format and used to select sequence reads containing these barcodes from the .bam files using samtools (v 0.1.19). The resulting .bam files were then converted to .fastq filed using bedtools (v 2.26.0) (Supplementary Fig. 1).

### HERV analysis

Processed fastq files were used to conduct our WHA tool (Supplementary Methods). Each sample was duplicated for the WHA analysis and then mapped with 3,220 HERV loci as reference data using BWA with minimum percent match > 99% (v 0.7.13). HERV loci that had 100% WSS scores were only selected as positive for the HERV loci. We defined a HERV loci transcriptome as negative if it had any HERV loci with a sequence depth of less than 3 or usable windows of less than 9. Conversely, HERV loci transcriptomes defined as positive had a sequence read depth greater than 3 and usable windows of greater than or equal to 9, corresponding to a minimum of 450 base pairs (i.e. 9 windows times 50 base pairs).

The HERV profiles between PBMC fresh and frozen samples from Derbois et al. were compared, and the resultant output was visualized in a Principal Component Analysis (PCA) plot using ClustVis [47]. To support the PCA plot, a phylogenetic tree was generated with the ‘normalize’ and ‘Euclidean’ options using R. A heatmap was also generated to display differences in HERV loci at across each sample using STAMP [48]. We also randomly subsampled sequence reads from both PBMC fresh and frozen samples and generated a PCA plot and phylogenetic tree to compare the results between the original reads and subsampled reads (Supplemental Fig. 2).

Additional manual filtering steps using the Excel files were applied across all the samples to determine HERV loci that were unique when all control samples were compared with either COVID-19 or MVC samples (Supplemental Table 8). To do this, only negative values were selected for the 21 control samples, and then at least one positive value remained for the COVID-19 or MVC samples. Then, the Venn diagrams were generated to represent the shared and unique HERV loci between (1) all controls vs. COVID-19, (2) all controls vs. MVC, (3) COVID-19 control vs. MVC, and (4) MVC control vs. COVID-19 using the gplots [49] and the VennDiagram packages in R [50].

### Statistical analysis

To determine significant differences in sequencing depth between control vs. either COVID-19 or MVC for each HERV loci, we conducted Mann-Whitney U test using R [51]. For this analysis, a *P* value of < 0.05 was considered statistically significant.

The positions of 3220 HERV proviral genomes in the human 23 chromosomes is depicted using *Idiographica* [52]. The individual HERV loci sequence identification can be found in Supplementary File 1.

A composite 21 normal individual pangenome control dataset was established using the control samples from the trauma, COVID-19 and an additional 5 normal PBMC samples [33] (Supplementary Table 4).

## Electronic supplementary material

Below is the link to the electronic supplementary material.

Supplementary Material 1

Supplementary Material 2

Supplementary Material 3

Supplementary Material 4

## Data Availability

The original sequencing data set of the samples used in this study was downloaded from the NCBI. (accession numbers: 1) 3 PBMC healthy individuals from Thompson, E.A., et al. (GSM5090446, GSM5090448, and GSM5090454); 2) 2 PBMC healthy individuals from Derbois et al. (GSM7077869 and GSM7077870); 3) 6 healthy individuals and 12 critical COVID-19 patients from Amrute et al. (GSM5746259, GSM5746260, GSM5746261, GSM5746262, GSM5746263, GSM5746264, GSM5746265, GSM5746266, GSM5746267, GSM5746268, GSM5746269, GSM5746270, GSM5746271, GSM5746272, GSM5746273, GSM5746274, GSM5746275, GSM5746276, GSM5746277, GSM5746278, GSM5746279, GSM5746280, GSM5746281, GSM5746282, GSM5746283, GSM5746284, GSM5746285, GSM5746286, GSM5746287, GSM5746288); 4) 10 healthy individuals and 10 trauma patients from Tianmeng et al. (GSM4960241, GSM4960242, GSM4960243, GSM4960245, GSM4960246, GSM4960256, GSM4960257, GSM4960258, GSM4960260, GSM4960261, GSM4960262, GSM4960271, GSM4960272, GSM4960273, GSM4960248, GSM4960249, GSM4960250, GSM4960253, GSM4960254, GSM4960264, GSM4960265, GSM4960267, GSM4960268, GSM4960269, GSM4960275, GSM4960276, GSM4960277, GSM4960244, GSM4960247, GSM4960251, GSM4960255, GSM4960259, GSM4960266, GSM4960263, GSM4960270, GSM4960274, GSM4960278)).
