# Supplementary material for: Shared and unique patterns of autonomous human endogenous retrovirus loci transcriptomes in CD14 + monocytes from individuals with physical trauma or infection with COVID-19
Source: Retrovirology. 2024 Nov 4;21:17. doi: 10.1186/s12977-024-00652-z (PMC11533341; doi:10.1186/s12977-024-00652-z)
Supplement: Supplementary file 1 — Supplementary Material 1 [file 12977_2024_652_MOESM1_ESM.docx]

**Supplemental Figure Legends**

**Supplemental Figure 1. Analysis pipeline for HERV loci transcriptomes.**

A depiction of the analysis pipeline is presented. For further details, see the main text.

**Supplemental Figure 2. Subsampled analysis of fresh versus frozen samples.**

To assure that clustering from the PCA presented in Figure 2 was not due to sample read differences, we repeated the analysis with equal sample read numbers (randomly sub-sampled 1, 2, and 3). We found no changes in phylogenetic tree and PCA clustering following rarefaction for each subsampling.

**Supplemental Figure 3. Sequence DNA read count versus number of positive HERV loci.**

The DNA sequences of HERV loci transcriptome expression patterns in the trauma or COVID-19 samples were compared with the composite 21 normal pangenome controls**.** Note that all individual composite pangenome control samples were filtered to be negative and are located on the axis. The individual samples from the trauma or COVID-19 datasets are presented on the Y-axis were negative. The individual positive HERV loci transcriptomes from the trauma and COVID study were plotted using the number of DNA sequence reads and the number of positives for each trauma or COVID-19 individual sample (**Supplemental Table 4)**. No correlation with the read counts were seen between the composite 21 genome pangenome control and positive samples in the trauma or COVID-19 samples (r^2^ = 0.29).
