# Supplementary material for: Shared and unique patterns of autonomous human endogenous retrovirus loci transcriptomes in CD14 + monocytes from individuals with physical trauma or infection with COVID-19
Source: Retrovirology. 2024 Nov 4;21:17. doi: 10.1186/s12977-024-00652-z (PMC11533341; doi:10.1186/s12977-024-00652-z)
Supplement: Supplementary file 3 — Supplementary Material 3 [file 12977_2024_652_MOESM3_ESM.docx]

Shared and unique patterns of autonomous human endogenous retrovirus loci transcriptomes in CD14+ monocytes from individuals with physical trauma or infection with COVID-19

Hyunmin Koo and Casey D. Morrow

**Supplementary Methods**

**Window-based SNV similarity (WSS) analysis.**

In this study, we used a WHA tool, which was modified from our previous tool, called WSS. For the WSS analysis, sequence reads from each sample were aligned to the 93 microbial reference genomes which were previously established based on the HMP dataset [1, 2] using the Burrows-Wheeler aligner (BWA) tool [3]. Each sample was analyzed for multi-sample SNVs relative to the provided reference genome using the Genome Analysis Toolkit (GATK) [4]. The resulting multi-sample Variant Call Format (VCF) files were utilized for pairwise comparisons between all possible pairs of samples. This was done to determine the overall genome-wide SNV similarity for each microbial species. Samples with sequence coverage below 30% and sequence depth less than 3.5 against their given reference genome were excluded from the pairwise comparisons [1, 5-11]. In order to identify related strains, the WSS score was compared to a previously established cut-off value from our earlier study. (For related strain pairs: WSS score>cut-off; for unrelated strain pairs: WSS score<cut-off) [1,12].

In WSS, a window is defined as similar if the SNV pattern is exactly the same between compared two samples or no SNV is present in both samples. Good (usable) windows are defined when each window had more than 50% of the bases having a satisfied coverage and depth value compared to cut-off values.

**Window-based HERV Alignment (WHA) analysis.**

In this study, we developed the Window-based HERV Alignment **(**WHA) analysis. Compared to the WSS, we replace the reference genomes from microbes with 3200 HERV loci. We also added new codes that can cluster cell-type specific hashtags and then filter the selected cell-type related sequence reads for pre-processing. Then, the same alignment tool was used to map HERV reference loci with different minimum percent matches, 99%. Each window size was adjusted from 100 base pairs to 50 base pairs, sequence depth cut-off was adjusted from 5 to 3, and a new cut-off value (total good/usable windows >8 representing at least 400 base pairs of DNA sequence) to identify positive HERV loci.

**References**

1. Kumar, R., et al., *Identification of donor microbe species that colonize and persist long term in the recipient after fecal transplant for recurrent Clostridium difficile.* NPJ biofilms and microbiomes, 2017. **3**(1): p. 12.

2. Schloissnig, S., et al., *Genomic variation landscape of the human gut microbiome.* Nature, 2013. **493**(7430): p. 45-50.

3. Li, H. and R. Durbin, *Fast and accurate long-read alignment with Burrows–Wheeler transform.* Bioinformatics, 2010. **26**(5): p. 589-595.

4. Van der Auwera, G.A., et al., *From FastQ data to high confidence variant calls: the Genome Analysis Toolkit best practices pipeline.* Curr Protoc Bioinformatics, 2013. **43**: p. 11.10.1-33.

5. Koo, H., D.K. Crossman, and C.D. Morrow, *Strain Tracking to Identify Individualized Patterns of Microbial Strain Stability in the Developing Infant Gut Ecosystem.* Frontiers in Pediatrics, 2020. **8**.

6. Koo, H., et al., *Individualized recovery of gut microbial strains post antibiotics.* NPJ Biofilms Microbiomes, 2019. **5**: p. 30.

7. Koo, H., et al., *Sharing of gut microbial strains between selected individual sets of twins cohabitating for decades.* PLOS One, 2019. **14**(12): p. e0226111.

8. Koo, H., et al., *An individualized mosaic of maternal microbial strains is transmitted to the infant gut microbial community.* Royal Society Open Science, 2020. **7**: p. 192200.

9. Koo, H. and C.D. Morrow, *Perturbation of the human gastrointestinal tract microbial ecosystem by oral drugs to treat chronic disease results in a spectrum of individual specific patterns of extinction and persistence of dominant microbial strains.* PLOS One, 2020. **15**(12): p. e0242021.

10. Koo, H. and C.D. Morrow, *Bacteroidales-specific antimicrobial gene analysis identifies gastrointestinal tract reservoirs of microbial sub strains selected for fecal dominance.* PREPRINT (Version 1) available at Research Square, 2022.

11. Koo, H. and C.D. Morrow, *Time series strain tracking analysis post fecal transplantation identifies individual specific patterns of fecal dominant donor, recipient, and unrelated microbial strains.* Plos one, 2022. **17**(9): p. e0274633.

12. Kumar, R., et al., *New microbe genomic variants in patients fecal community following surgical disruption of the upper human gastrointestinal tract.* Human Microbiome Journal, 2018. **10**: p. 37-42.
