# Supplementary material for: Shared and unique patterns of autonomous human endogenous retrovirus loci transcriptomes in CD14 + monocytes from individuals with physical trauma or infection with COVID-19
Source: Retrovirology. 2024 Nov 4;21:17. doi: 10.1186/s12977-024-00652-z (PMC11533341; doi:10.1186/s12977-024-00652-z)

Supplementary Figure 1

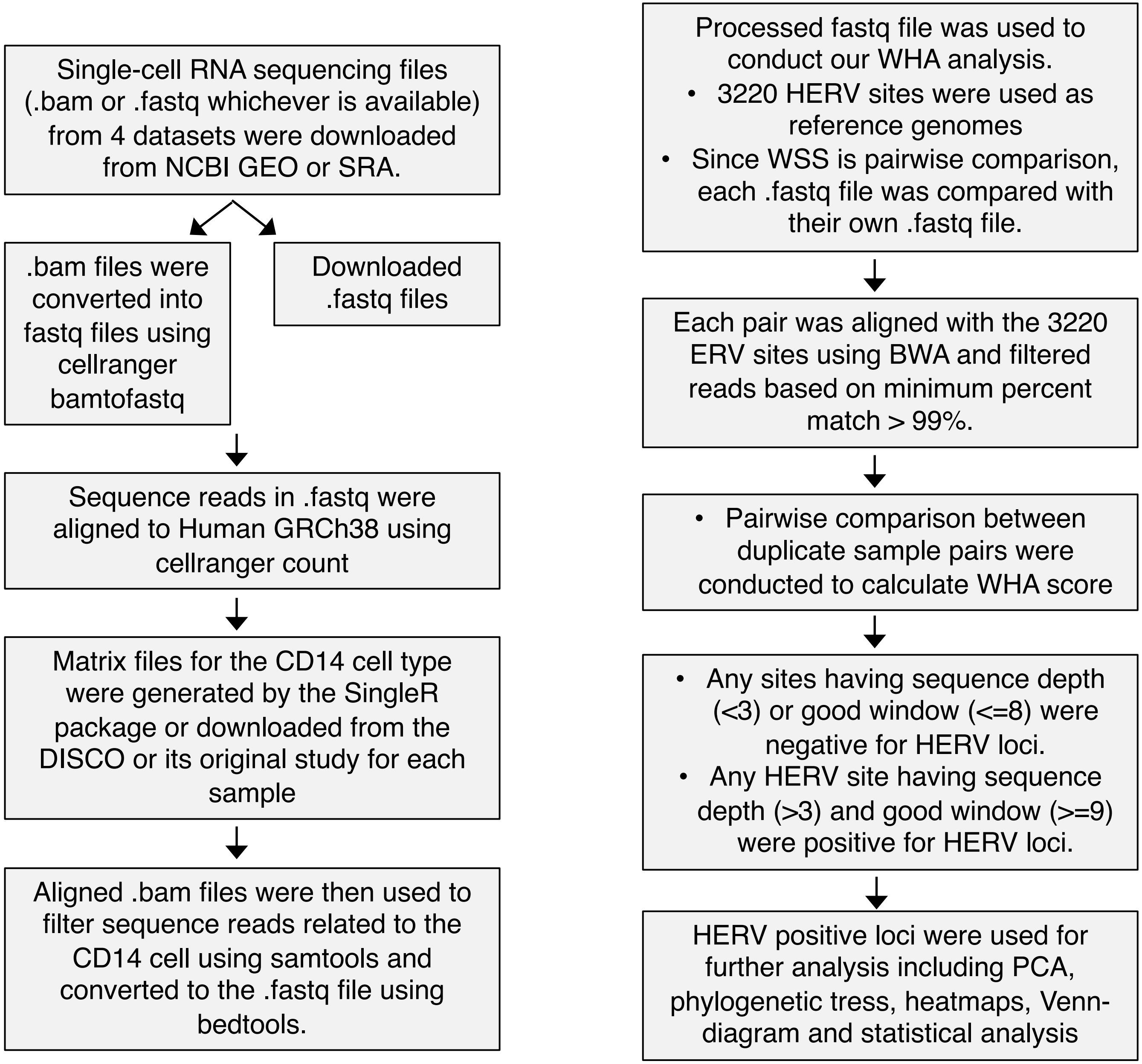

Supplementary Figure 2 (1st random subsampling)

(A)

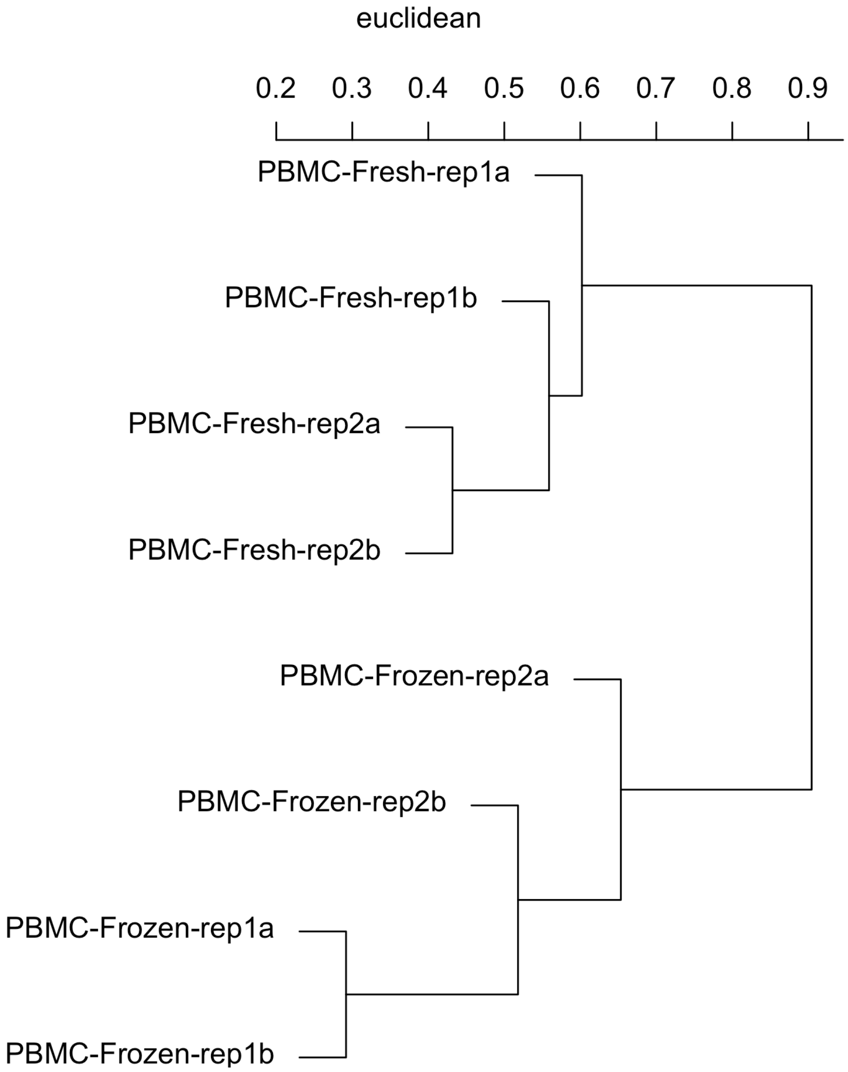

(B)

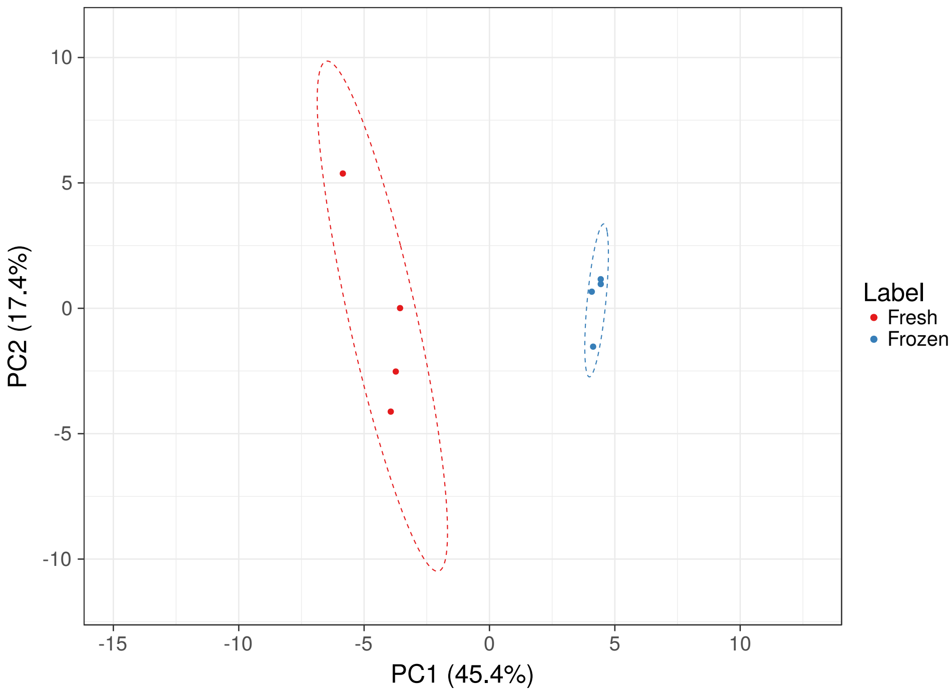

Supplementary Figure 2 (2nd random subsampling)

(A)

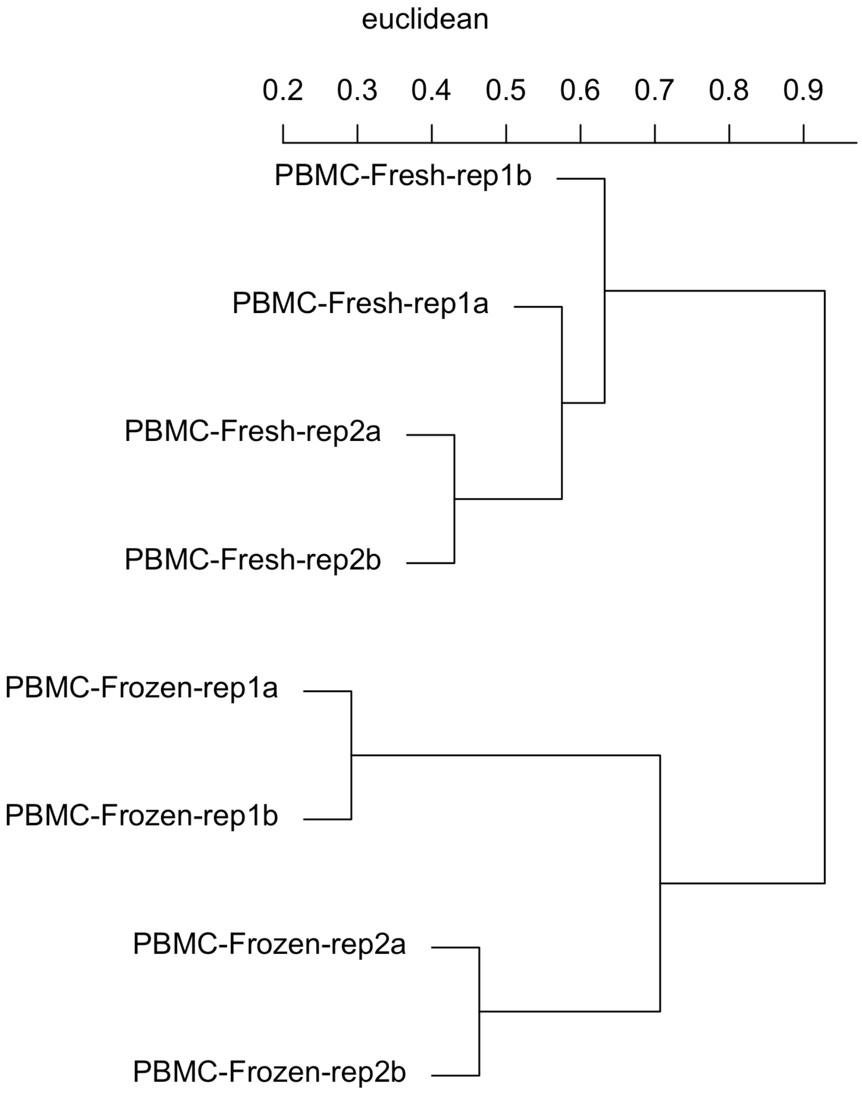

(B)

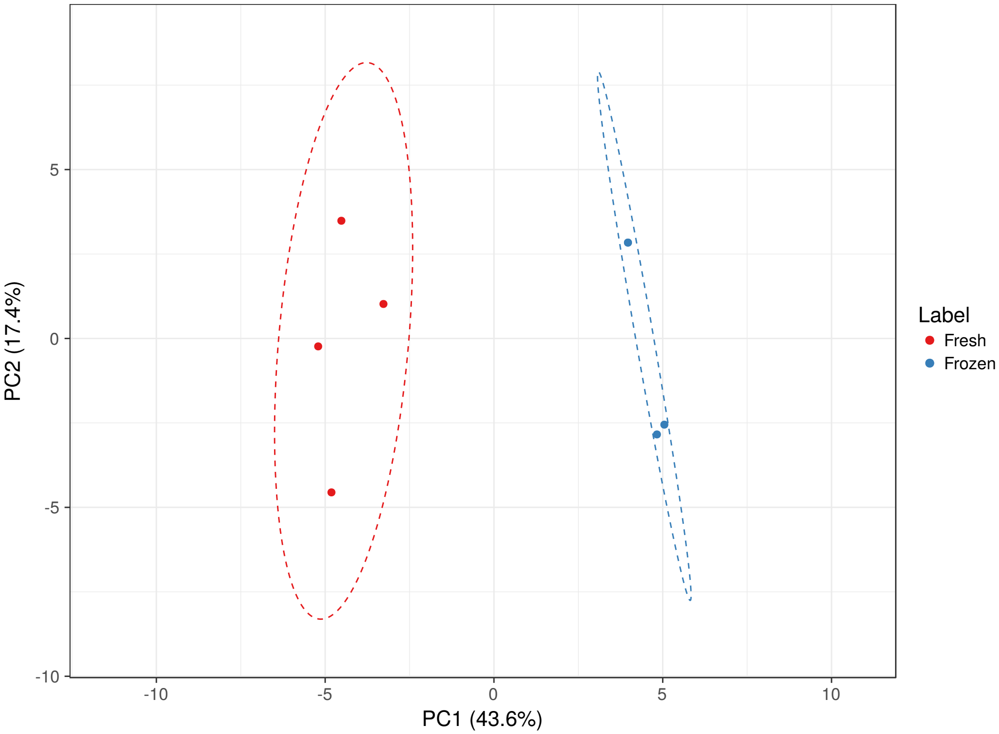

Supplementary Figure 2 (3rd random subsampling)

(A)

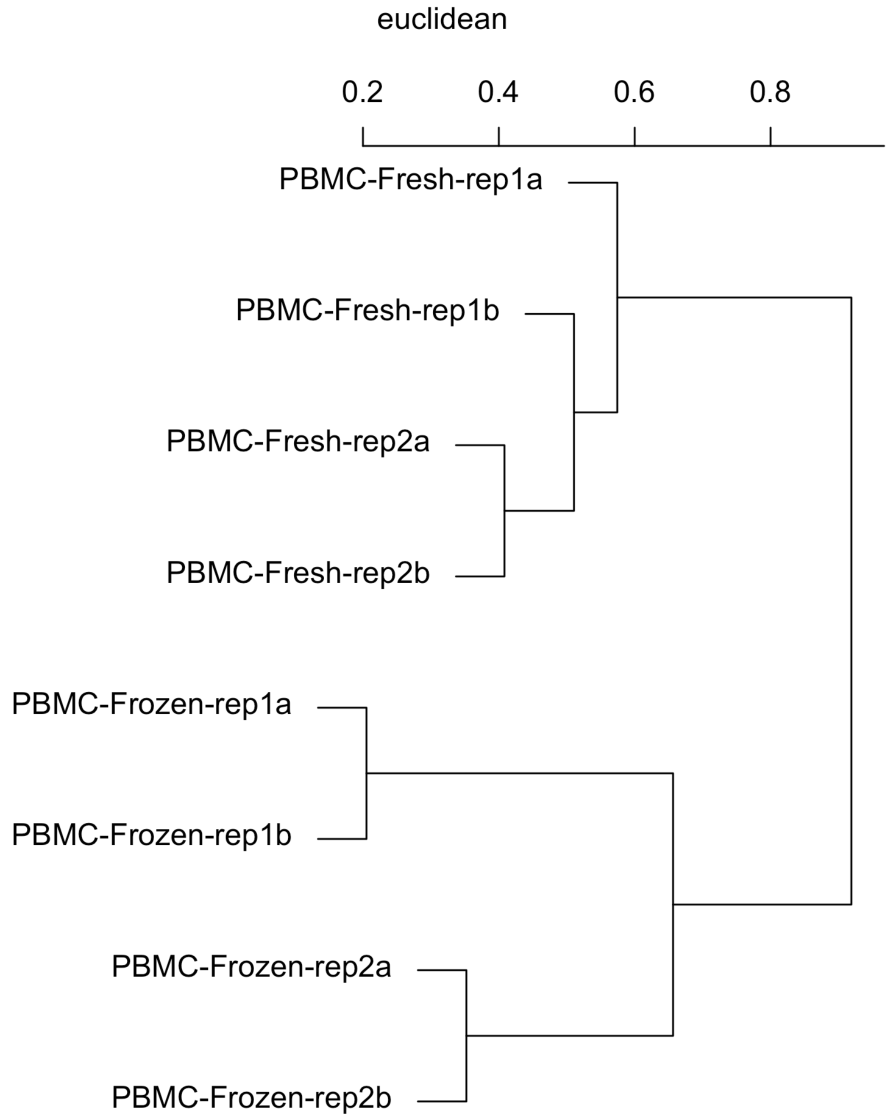

(B)

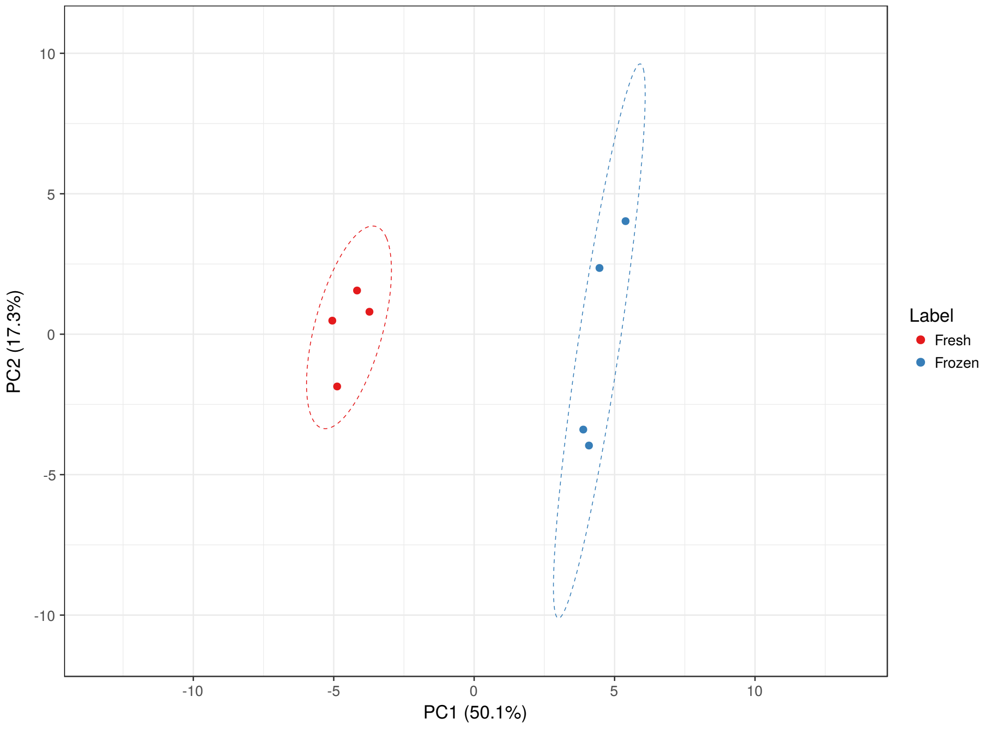

Supplementary Figure 3

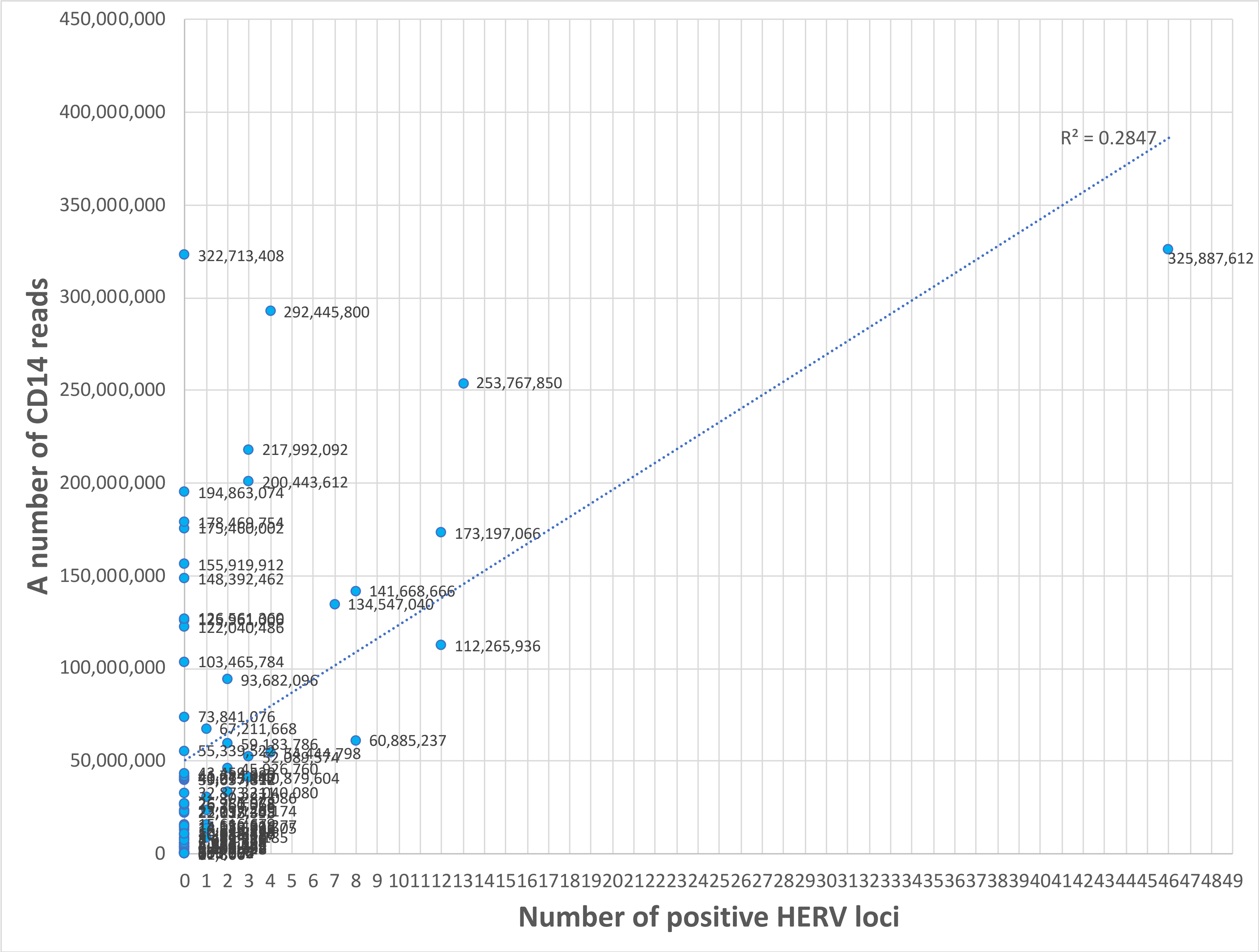

Supplement: Supplementary file 4 — Supplementary Material 4 [file 12977_2024_652_MOESM4_ESM.pdf]
